# VariantDB: a flexible annotation and filtering portal for next generation sequencing data

**DOI:** 10.1186/s13073-014-0074-6

**Published:** 2014-10-02

**Authors:** Geert Vandeweyer, Lut Van Laer, Bart Loeys, Tim Van den Bulcke, R Frank Kooy

**Affiliations:** Department of Medical Genetics, University of Antwerp, 2650 Edegem, Antwerp Belgium; Biomedical Informatics Research Center Antwerp, University and University Hospital of Antwerp, 2650 Edegem, Antwerp Belgium; Department of Medical Genetics, University Hospital of Antwerp, 2650 Edegem, Antwerp Belgium

## Abstract

Interpretation of the multitude of variants obtained from next generation sequencing (NGS) is labor intensive and complex. Web-based interfaces such as Galaxy streamline the generation of variant lists but lack flexibility in the downstream annotation and filtering that are necessary to identify causative variants in medical genomics. To this end, we built VariantDB, a web-based interactive annotation and filtering platform that automatically annotates variants with allele frequencies, functional impact, pathogenicity predictions and pathway information. VariantDB allows filtering by all annotations, under dominant, recessive or *de novo* inheritance models and is freely available at http://www.biomina.be/app/variantdb/.

**Electronic supplementary material:**

The online version of this article (doi:10.1186/s13073-014-0074-6) contains supplementary material, which is available to authorized users.

## Background

Next generation sequencing (NGS) has the power to screen a whole genome for all kinds of genetic variation in a single experiment [1]. In medical genetics, NGS has proven to be a key tool to identify disease-causing mutations in individuals with Mendelian disorders. Most studies so far have concentrated on the exome or protein coding part of the genome, which comprises only 1.5% of the complete human genome. Despite the smaller target size, whole exome sequencing (WES) typically yields over 20,000 protein altering variants per sample [2,3]. Today, several studies have proven the potential of WES to identify causal genetic defects underlying various disorders in a substantial number of patients [4-6]. As such, WES greatly reduces experimental costs while achieving high analytical power. Despite the proven utility of, and high diagnostic demand for, NGS-based assays, interpretation and filtering of the extensive variant lists is currently a labor-intensive and cumbersome task, and hampers the implementation of WES in routine diagnostics [3,4].

NGS data analysis can be subdivided into two sequential subtasks. The first task comprises quality control of the raw sequencing reads, mapping reads to a reference genome and generating a primary variant list [7]. The second stage comprises interpretation of the variants in relation to the patient’s phenotype. Several approaches are available to handle the read-to-variant stage. Commercial packages often offer all-in-one solutions such as SeqNext [8], CLCBio Genomic Workbench [9] or Illumina’s CASAVA [10]. Academic solutions on the other hand typically consist of the combination of sequential tools for specific steps in the analysis. These include tools for cleaning up the sequence (for example, FASTX-Toolkit [11], CutAdapt [12]), aligning reads to the genome (for example, Bowtie [13], BWA [14]) and variant calling (for example, samtools [15], Genome Analysis Toolkit (GATK) [16]). Out of this extensive collection of analysis options, the research community has converged on a BWA-GATK based pipeline as the preferred method, as it appears to have the highest sensitivity and specificity. Recently, the superiority of this consensus approach was corroborated by an in-depth performance analysis of several available methods [17]. Galaxy, a flexible and publicly available online platform, offers streamlined execution of consecutive processing steps to non-bioinformatics experts, thus providing a straightforward implementation of the first analysis stage [18-20].

Ideally, the second analysis stage would be able to handle identified variants of either a single sample, a family-based analysis, or a case/control study, while at the same time integrating extensive annotation with biological information and dynamic filtering. Commercial packages such as Bench Suite [21] provide turn-key solutions for variant annotation, interpretation and prioritization. However, these platforms are tailored at long-term usage in routine clinical diagnostics laboratories, and are less suitable for use in smaller laboratories or research settings that typically demand more flexible and less expensive solutions.

Currently available academic software still requires the manual inspection of variants using a combination of web tools and stand-alone packages. Many of these tools were developed for specific research questions, such as either family-based [22,23] or case/control-based experiments [24], or provide broad annotation in text-based output without dynamic filtering options [23,25-28]. Other available tools provide dynamic filtering options but can only handle a limited set of annotations [29-31]. Direct integration of the first and second analysis stage, bypassing manual handling of intermediate results, is a feature currently only available in the WEP platform [32]. Finally, as both genetic and phenotypic heterogeneity appear to be an emerging theme in many genetic disorders, it is clear that WES data should be evaluated in the context of large cohorts of patients and controls [33]. Hence, online collaboration between genetic centers in a protected setting, which is available only for a limited number of current tools, provides a significant advantage [29].

To overcome the limitations of currently available solutions in the complex annotation and filtering stage of NGS data analysis, we developed VariantDB. It unifies broad annotation and flexible filtering strategies in a user-friendly online interface and at the same time provides direct integration with the semi-automatic analysis capabilities of platforms such as Galaxy. Furthermore, it allows collaboration and data protection using role-based authentication.

## Implementation

### Interface and database

**Figure 1:**
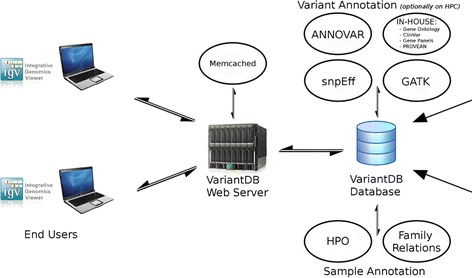
**Schematic representation of VariantDB implementation.** Depending on the expected platform load, server elements can be hosted either on a single machine (default) or on separate physical hosts. If high performance computing (HPC) infrastructure is available, annotation processes can be distributed. HPO, Human Phenotype Ontology.

VariantDB consists of a PHP (5.3.2) based web interface, driving a CGI (5.10.1) backend. All data are stored in a MySQL (5.1.41) database on solid state drives (Figure 1). Structurally, data are ordered in sample and variant specific tables (Additional file 1). One additional table links variants to samples and holds quality information from GATK. Variant annotations are stored in separate tables based on the annotation source. This structure optionally allows VariantDB to retrieve annotation or filtering data from multiple sources in parallel, using the Perl Parallel::ForkManager library. Further improvements in performance can be achieved by enabling Memcached. The Perl Cache::Memcached::Fast library can reduce database load by caching and preloading frequently used data in memory. Queries, sources, and documentation for all filters and annotations are stored in XML files. Additional filtering rules can be specified as separate nodes in these configuration files.

A public VariantDB instance is available for academic use. Furthermore, local installation is supported through either a downloadable virtualbox application or full installation on local infrastructure. Instructions for both approaches are available in the online documentation. To keep local installations up to date, automatic updating through the web interface is possible for the local administrator.

### Data import

VCF files can be imported from an FTP server, accessible using VariantDB user credentials, or directly from a Galaxy server using the VariantDB tool (Additional file 2; for installation see [34]). Imported VCF files should comply with the VCF4.0 standards. Quality annotations generated by the GATK-based genotypers [7] are extracted and stored.

VariantDB provides the option to store the imported VCF file and associated BAM file. If available, direct links are presented to load VCF and BAM files into Integrative Genomics Viewer (IGV) for visualization of filtering results [35].

### Annotation

**Figure 2:**
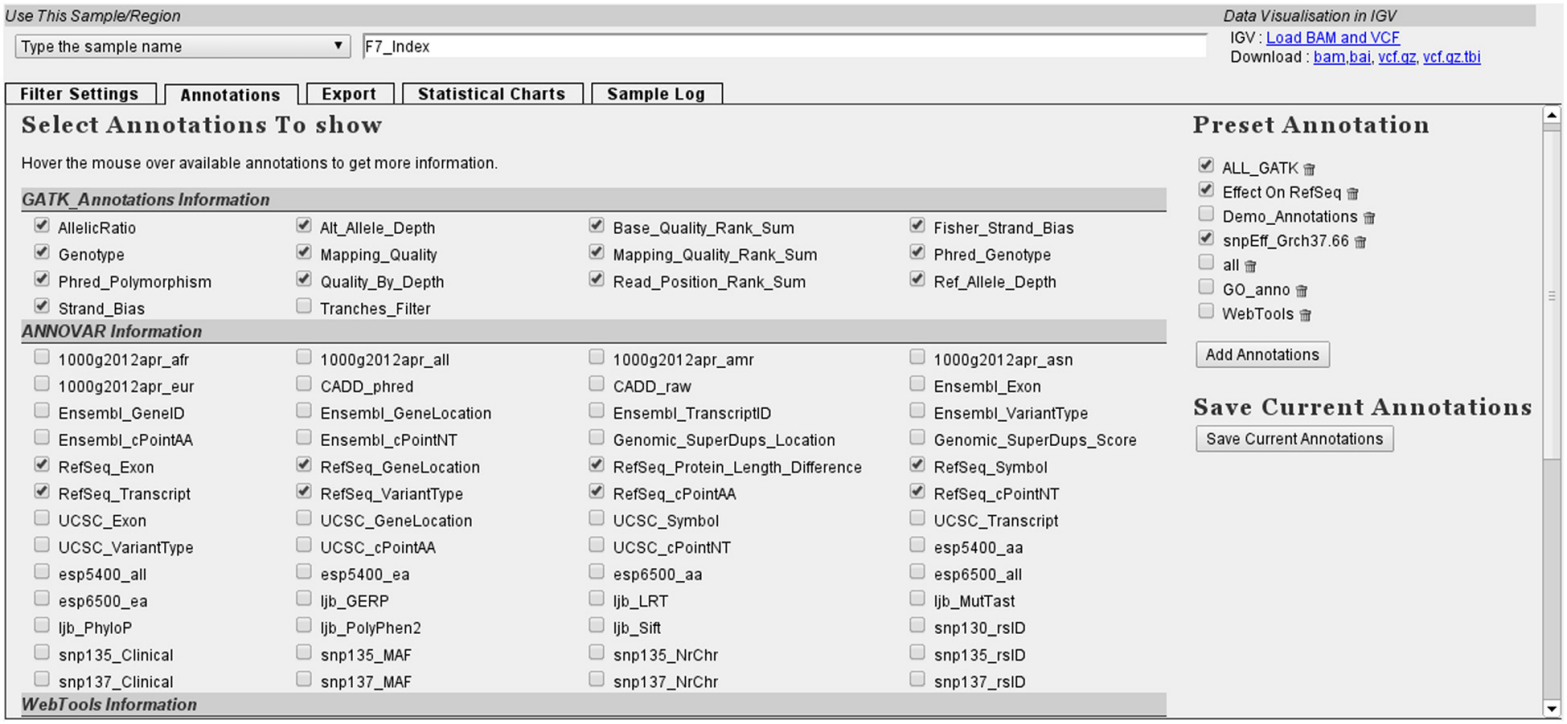
**Selection of annotations.** Top left: sample selection box, using either a dropdown menu, or auto-completion. Top right: when raw data files are available, hyperlinks are presented to download VCF/BAM files or load the files into IGV. Bottom left: all available annotations are listed. Users can select annotations using checkboxes for inclusion into the filtering results. Bottom right: previously saved sets of annotations can be enabled at once by selecting the checkbox and pressing ‘Add Annotations’.

Data annotation within VariantDB is available at sample and variant levels. With regard to sample annotation, family and experimental relations can be provided, which can later be applied to formulate inheritance patterns for variant filtering. Second, gender and phenotype information based on the Human Phenotype Ontology [36] is available. Finally, samples can be labeled as controls, which allow exclusion of common variants in filtering. Variant annotation is triggered by importing VCF files. Annotation proceeds by collecting variants missing a respective annotation, annotating the list of variants, and storing the results in the database. The annotation-specific tables in the database structure allow this process to be parallelized. If a high performance computing infrastructure is available, VariantDB can be configured to distribute these processes using the Perl Schedule::DRMAAc module (0.81). In total, 110 annotations are added to each variant (Table 1), taken from eight sources. The annotation engine utilizes ANNOVAR, snpEff, the Perl WWW::Mechanize library (for web tools) and a set of in-house parsers to retrieve the annotations [25,28]. All annotations are presented by checkboxes in VariantDB for inclusion into the results (Figure 2). Users can also define sets of annotations that can be loaded simultaneously.

**Table 1:** **Summary of annotations available in VariantDB**

**Source tool**	**Available annotations**	**Reference**
GATK genotypers	Variant coverage, allelic ratio, genotype, Phred polymorphism, Phred genotype, quality by depth, mapping quality, ranksums, strand bias	[16]
ANNOVAR	Allele frequencies (1KG/ESP/dbSNP), pathogenicity (dbNSFP, CADD, GERP++), segdups, genes (symbol, exon, location, effect; UCSC/RefGene/Ensembl)	[28]
SnpEff	Variant effect, effect impact, location, protein change, gene (Ensembl)	[25]
Web tools	MutationTaster, SIFT, PROVEAN, Grantham	[37-39]
Gene Ontology	Associated Gene Ontology IDs, terms, and term types. First level parental terms	[40]
ClinVar	Link to ClinVar, variant type, pathogenic class, class comment, affected gene and transcript, latest update, associated disease, links to external data sources, publications	[41]
Gene panels	Affected gene, comments, panel name

GATK genotyping modules provide a set of quality parameters for each identified variant. VariantDB stores the values of the allelic ratio, Phred score of the polymorphism (QUAL), Phred-based genotype quality (GQ), genotype (GT), allelic depths (AD), quality by depth (QD), mapping quality (MQ), strand bias (FS) and rank sums (BaseQRankSum, MQRankSum, ReadPosRankSum). If available, filter entries such as the VQSR tranches filter, are also stored.

Minor allele frequencies (MAFs) are available from the 1000 Genomes Project (v.2012apr) and the exome sequencing project (v.esp5400.2012Jul11, v.esp6500.2013Jan22), both global and population specific [42,43]. Second, dbSNP rsIDs, MAFs and population size values are available for versions 130, 135 and 137 [44]. Starting from version 135, the clinical association label is also extracted.

Transcript information is extracted in UCSC, RefSeq and Ensembl-based format. Available information includes gene symbol or ID, transcript ID in case of multiple variants, affected position on cDNA and protein level and the effect on the protein level (intron/exon, missense/synonymous/nonsense, splicing).

Predictions with regard to pathogenicity are included from several tools. Using ANNOVAR, dbSNFP annotations for LRT, MutationTaster, PhyloP, PolyPhen2 and SIFT are included [45]. GERP++ [46] and CADD [47] scores are added from the respective tool data. Up-to-date scores of PROVEAN, SIFT, Grantham and MutationTaster are retrieved using the respective web tools [37,38]. Finally, the SnpEff annotations also provide an estimate of the variant impact on the protein function [25].

Two sources are provided for functional annotation. First, Gene Ontology terms and the first level parental terms associated with affected genes are provided [40]. Second, a summary of the information available in ClinVar is available [41]. This summary includes hyperlinks to the ClinVar entry of variants that exactly match or overlap the variant in the queried sample, the type of variant in ClinVar (SNP/indel), the affected gene and transcript, latest update, evidence type, pathogenicity classification and associated disease. For gene, disease and alleles listed in ClinVar, hyperlinks are provided to several external databases.

Finally, users can specify additional information on inheritance, experimental validation and diagnostic classification on a per variant level.

### Annotation updates

VariantDB provides two functionality layers to automatically keep annotation sources up to date. First, using scheduled execution at a frequency specified by the system administrator, third-party resources are checked for updated releases. When new data are available, all variants are re-annotated using the new release. To maintain data traceability, all discarded annotations are archived and all changes to variant annotation are logged. Finally, users are informed by email of possibly relevant novel annotations. Second, VariantDB automates the conversion between genome builds from the web interface. Upon conversion, the platform administrator needs information on the new build, including ANNOVAR, snpEff and IGV genome versions (hg19, GRC37.66 and hg19, respectively, for the current VariantDB version). Availability of the requested build is checked and, if available, all annotation tables are downloaded. Genome coordinates of currently stored variants are converted using the UCSC LiftOver tool, and failed conversions are presented to the platform administrator for manual curation [48]. Finally, all variants are re-annotated with regard to the new coordinates and users are informed. Previous genome versions remain accessible with their final annotations in read-only mode. The current genome build is always stated in the user interface. Also, when importing data from external pipelines such as galaxy, VariantDB requires the source genome build version to be passed along with the variant files, and will generate an error message on conflicting versions.

### Variant filtering

**Figure 3:**
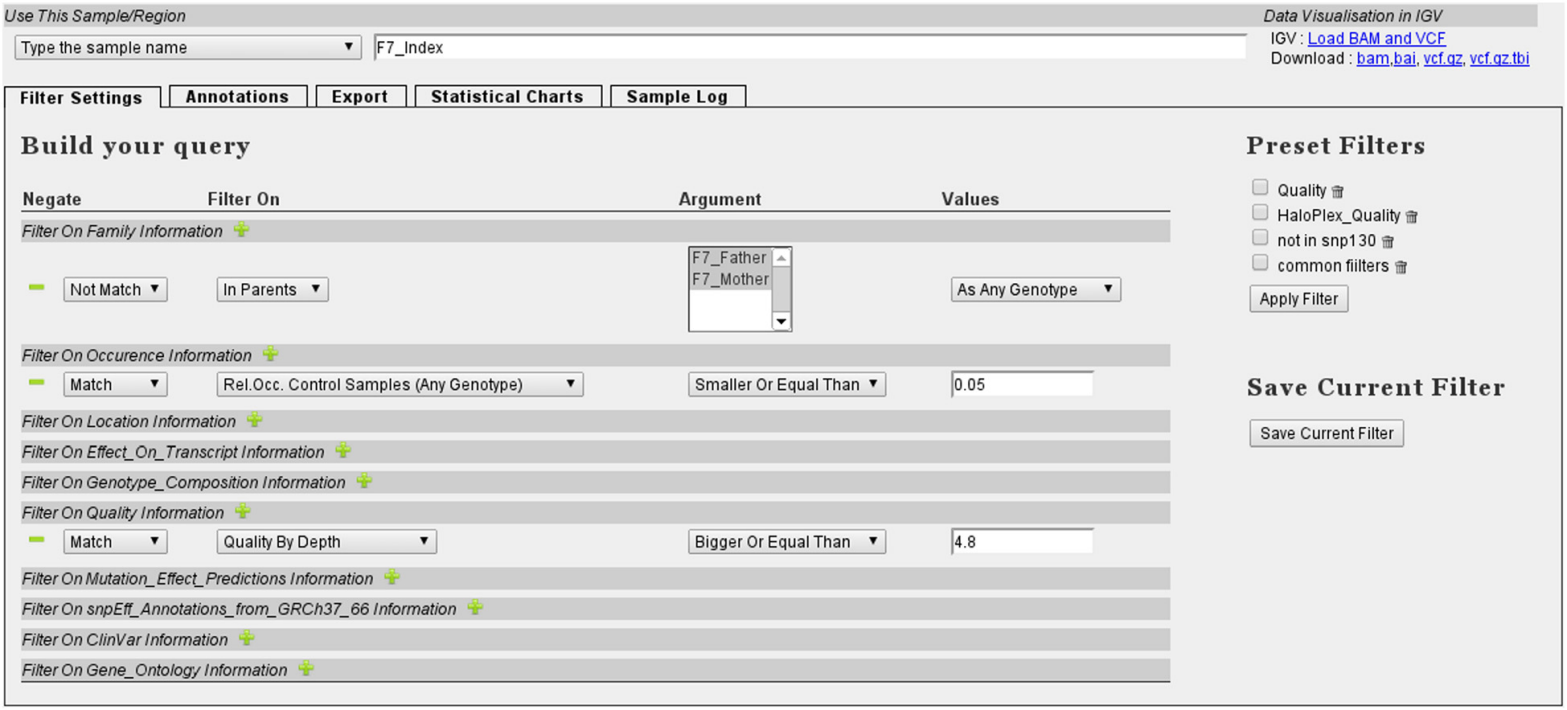
**Selection of filters.** Left: filtering criteria are organized in high-level categories. Filters are added by selecting the relevant filter and settings from dropdown menus. Numeric (for example, quality control values) or textual (for example, Gene Symbol) criteria can be added in text fields where appropriate. Right: previously saved filtering schemes can be enabled at once by selecting the checkbox and pressing ‘Apply Filter’.

**Figure 4:**
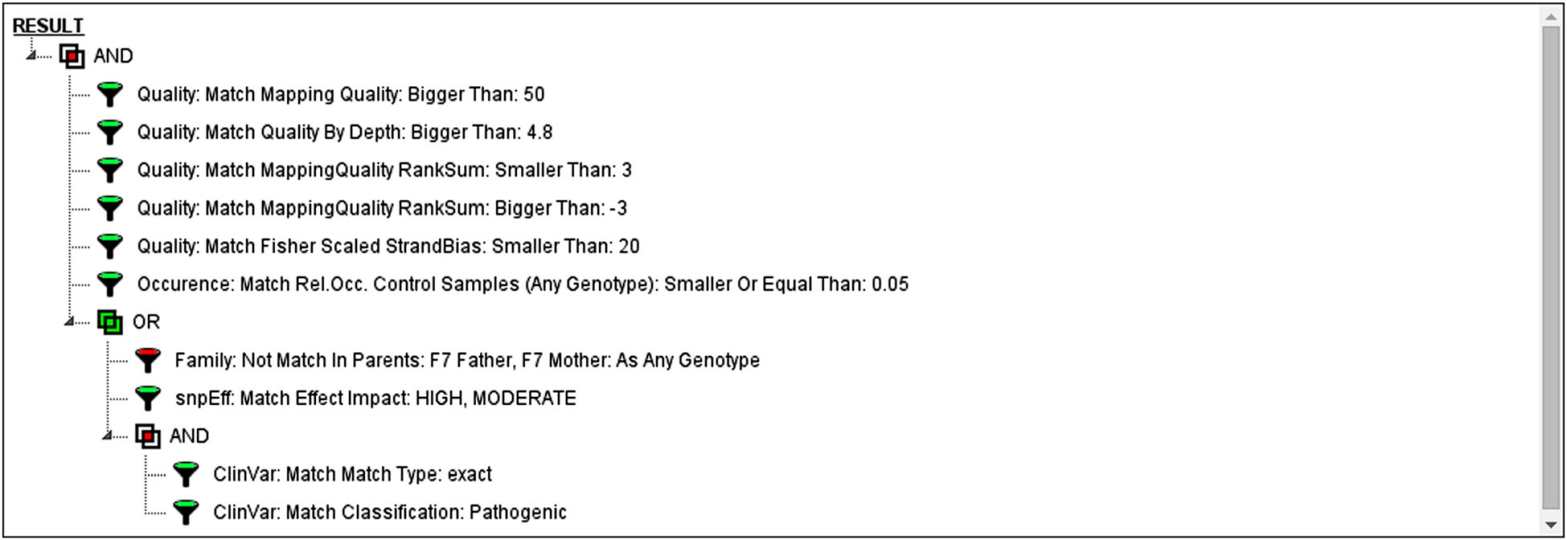
**Graphical representation of the selected filtering scheme.** Individual filters can be grouped using logic AND/OR rules. Grouping and ordering is handled using a drag-and-drop interface.

VariantDB allows filtering on a combination of any of the available annotations listed in Table 1. To set filters, users select the criteria from dropdown menus (Figure 3) and optionally group them into a multi-level decision scheme (Figure 4). Successful filter settings can be saved for future usage. Next to the functional filtering criteria, parental and sibling relationships enable filtering for *de novo*, dominant and recessive inheritance models. Population-based variant selection can be performed on two levels. First, users can select variants that are present at least, or no more than, a specified number of times in a selection of samples. Second, genes can be selected for mutation burden by specifying the minimal or maximal number of samples containing a mutation in the same gene.

Next to general gene and population level information, users can create *in silico* gene panels for targeted evaluation of candidate genes. A gene panel exists of a set of RefSeq identifiers, optionally augmented with additional comments. Gene panels are private at the user level, but can be made available as a public resource to all users.

### Visualisation

**Figure 5:**
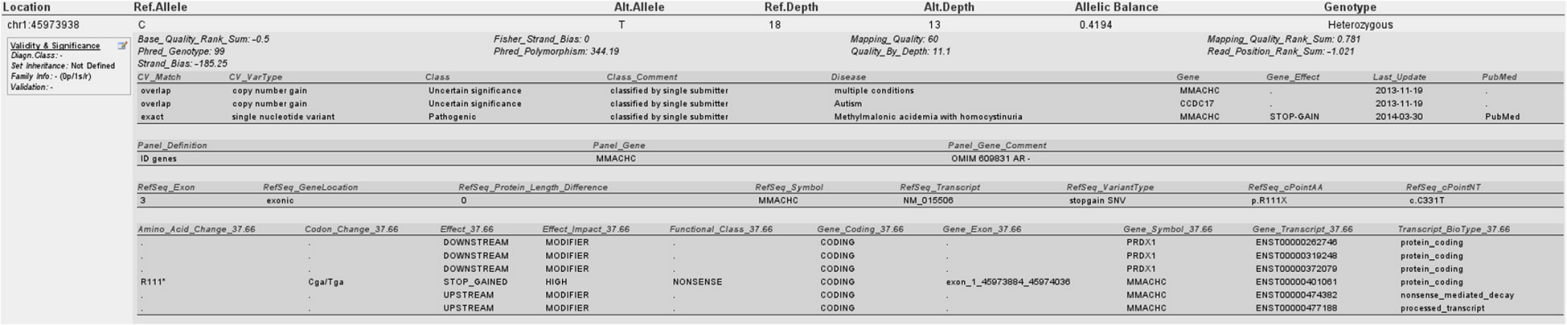
**Results table.** For each of the resulting variants, selected annotations are presented. On top, genomic position (which is also a hyperlink to the position in IGV), and other essential variant information is provided. If relevant, annotations are grouped in sub-tables on affected feature. User-specified information related to validation and classification is presented in a separate box on the left-hand side.

By default, results are presented in a tabular overview (Figure 5) with selected annotations and IGV hyperlinks [35]. VariantDB aims at presenting all information related to a variant in a compact single screen view. Alternatively, a classic, wide table format is available, presenting all annotations on a single line per variant (Additional file 3). Results can also be exported to CSV files for downstream analysis. Finally, various charts are available to review the quality or characteristics of the resulting variant set. These charts include, among others, the Tr/Tv ratio, known versus novel ratio, MAF distribution and SNP versus indel ratio.

## Results and discussion

### Integration with existing NGS data processing systems

VariantDB provides a broad annotation of the detected variants, in combination with relevant filtering schemes and seamless integration with upstream data processing by means of a dedicated Galaxy tool. Communication between Galaxy and VariantDB occurs through generic HTTP-based forms. Hence, import of VCF files into VariantDB can be implemented as the endpoint of any NGS data analysis pipeline running on high performance computing infrastructure with internet access. We have chosen to support data import for VCF files only, as this format is the current community standard for NGS data. Although any generic VCF file can be loaded into VariantDB, GATK-based variant calling (Unified Genotyper, Haplotype Caller, MuTect [16,49]) is currently regarded as the gold standard [17]. Therefore, we included specific import of various quality scores from GATK-based VCF files.

### Filtering approaches

In total 110 annotations are available targeting specific aspects for selecting relevant variants. Although all annotations can be used as filtering criteria, two of the main approaches are gene-based and family/cohort-based filtering.

Gene-centric information is provided according to NCBI, Ensembl and UCSC nomenclature. To guarantee optimal sensitivity, filters to select variants that affect exonic sequence (Gene Location filter) or lead to a premature stop codon (VariantType filter) are applied in a transcript-specific manner. Using this approach, all genes where a variant introduces a stop codon in at least one transcript variant are reported. Apart from unbiased filtering, users can specify a list of candidate genes to perform *in silico* targeted analysis (Location Information filter). *In silico* gene panel analysis offers a two-step analysis for molecular diagnostics. By reducing the risk of incidental findings in initial analysis, a two-step approach lowers psychological distress for patients undergoing genetic testing [50]. If no causal variants are found in the candidate genes, whole exome or whole genome data are still available for follow-up investigation.

When family or cohort information is available, this information can be used to further refine the variant list. As an example, in a recessive disorder one would select homozygous variants (Genotype Composition filter) in a patient, which are present as heterozygous variants in both parents (Family Information filter). In the absence of such information, VariantDB can select for rare variants based on MAFs taken from dbSNP, the 1000 Genomes Project, the Exome Sequencing Project, or a private control cohort (Occurrence Information filter).

### Ascertaining biological relevance

Although a selected filtering approach might already imply a certain biological relevance of the resulting variants (for example, *de novo* stop mutations), specific annotations are provided in VariantDB to further interpret the effect of a variant. First, known clinical associations are available in dbSNP as of version v135. More extensive information, however, is added from ClinVar (Clinvar Information filter) [41]. This database brings together genotype and phenotype data for known genetic variants, both SNP and structural variants, together with experimental data, links to external resources and relevant literature. Since its release in 2012, ClinVar rapidly became a reference resource for the interpretation of high throughput genetic data [51]. Second, information on the biological function of affected genes is presented based on Gene Ontology [40].

Finally, several prediction algorithms are available within VariantDB for the ascertainment of the variant pathogenicity (Mutation Effect Prediction filter). These predictions are typically based on evolutionary conservation [37,39,52], biochemical properties of the altered amino acids [53], or a combination of these [38,54]. CADD, a novel prediction algorithm, was recently described and added to VariantDB. It integrates over 60 different annotations into a single model for variant deleteriousness, showing a significantly higher performance than previous methods [47]. With ClinVar and CADD, VariantDB thus contains two state-of-the-art annotation resources to interpret the functional impact of variants, in addition to several other widely used annotation sources.

### Retrospective analysis

The development of various high-throughput screening methods resulted in an ever increasing amount of biological knowledge. Due to the continuously evolving interpretational resources, researchers are faced with the need to periodically reevaluate previous experiments for novel insights. VariantDB is, to our knowledge, the only publicly available platform that has the functionality to automatically handle such retrospective analyses. It updates all third-party resources on a preset time schedule, and notifies users when novel putatively interesting annotations are available. Here, we define putatively interesting as variants with a potential high impact on protein function (for example, frameshift or nonsense), based on both the RefSeq and the more comprehensive Ensembl gene sets, or matching variants classified as clinically relevant in ClinVar.

### Performance

At the time of writing, the public VariantDB server holds over 46 million variants from almost 2,000 samples, corresponding to 2.2 million unique variants. By utilizing data caching and pre-fetching of data while users are setting filters, we achieve sufficient performance to allow interactive filtering and annotation of results (Table 2). After filtering, results are presented in batches of 100 variants to the user (Figure 5).

**Table 2:** **Performance examples of VariantDB**

**Sample**	**Filters**	**Number of resulting variants**	**Number of annotations**	**First run** ^**a**^	**Second run** ^**b**^
Exome (77 K variants)	*De novo*, exonic , five quality thresholds	859	31	8 s	6 s
Exome (78 K variants)	Five quality thresholds, SnpEff high/moderate impact	1,007	110	14 s	8 s
Exome (78 K variants)	None^c^	78,423	110	12 s	11 s

^a^Results are retrieved from the database, and cached for future use. ^b^Results are retrieved from cache. ^c^No filters are specified. As only the first 100 variants, ordered by genomic position, are initially presented, runtime is not significantly larger.

### Data protection

VariantDB contains a user authentication module to protect stored data. Projects, defined as a collection of samples, can be shared with collaborators with rights ranging from read-only access to the ability to edit or delete whole projects. This online, role-based approach offers a major advantage over desktop solutions such as VarSifter or PriVar, and web-based but single-user approaches such as EVA [30,31,55]. As a centralized solution, VariantDB enables intuitive retrospective or multi-sample analysis, and collaboration between researchers from multiple laboratories. This was already successfully demonstrated in multiple published and ongoing studies [33,56-58] (Proost *et al.*, Sommen *et al.*, unpublished results).

For an institutional setup of VariantDB, we provide private installation of the platform behind local firewalls. This can either be the deployment of a preinstalled virtual machine or full installation on private infrastructure.

## Conclusions

VariantDB offers an all-in-one solution for annotation and filtering of variants obtained from NGS experiments. As summarized in Table 3, all the currently available platforms lack one or more of the essential aspects of variant interpretation present in VariantDB. It combines a broad range of annotations and filters, thereby eliminating the need for bioinformatics expertise by the user. Availability of *in silico* gene panel analysis reduces the risk of incidental findings, while centralized data storage enables large multi-center study designs, automated and retrospective updates of annotations and data traceability. The modularity of VariantDB offers extensibility with field-specific (for example, COSMIC for cancer research) and future (for example, ENCODE for whole genome sequencing interpretation) annotations and annotation tools in local instances. Overall, we conclude that VariantDB has a significant added value in streamlining NGS data analysis.

**Table 3:** **Functional comparison of VariantDB with publicly available alternatives**

	**KggSeq**	**VariantMaster**	**BIERapp**	**AnsNGS**	**WEP**	**FamANN**	**PriVar**	**EVA**	**Annotate-it**	**VariantDB**
Citation	[59]	[60]	[61]	[62]	[32]	[23]	[30]	[31]	[29]	
**Data management**										
Online	-	-	+	+	+	-	-	+	+	+
Collaborative projects	-	-	-	-	+	-	-	+	+	+
Inter-sample relations^a^	+	+	+	-	+	+	+	+	+	+
**Gene annotations**										
RefSeq annotations	+	+	+	+	+	+	+	+	+	+
Ensembl annotations	-	+	+	-	-	+	-	-	+	+
*In silico* gene panels	-	-	+	-	-	-	+	-	+	+
**Population frequencies**										
Public (ESP, 1KG, dbSNP)	+	+	+	-	+	+	+	+	+	+
In-house samples^b^	+	-	-	-	-	-	-	-	-	+
**Pathogenicity predictions**										
dbNSFP^(c)^	+	-	+	-	+	+	+	-	+	+
CADD	-	-	-	-	-	-	-	-	-	+
PROVEAN	-	-	-	-	-	-	-	-	-	+
**Clincal**										
Disease information source	GSEA	-	ClinVar	MIM	-	-	HuGe	-	MIM	ClinVar
**System implementation**										
Annotation updates^d^	A	M	A	.	.	M	M	M	A	A
Retrospective updates	-	-	-	-	.	-	-	-	-	+
Upstream integration^e^	-	-	-	-	+	+	-	-	-	+
Alignment visualization	-	-	+	-	+	-	-	-	-	+

^a^Relations might be either specified at sample level or provided as pedigree files upon runtime. ^b^User-accessible sample genotypes are used to calculate a private set of MAFs. ^c^Both full and partial dbNSFP annotations are considered here. ^d^A, automatic; M, manual annotation updates; or not specified (period). ^e^Direct integration with genotyping tools or modules.

## Availability and requirements

- **Project Name:** VariantDB
- **Project homepage:**http://www.biomina.be/app/variantdb
- **Operating system:** Ubuntu Linux
- **Programming language:** Perl, php/cgi
- **License:** GPLv3
- **Restrictions for non-academics:** ANNOVAR license needed

## Additional files

Additional file 1:
**Database layout of VariantDB.** The variant table holds unique IDs and information on genomic position and allelic composition of each observed variant. Annotations are stored in separate tables linked on the variant_id. To increase performance, ClinVar and Gene Ontology are stored as summary representations of the full releases. User authentication is based on email and password combinations. Data access is controlled on the project level, with access definitions for variant-level, sample-level and phenotype-level editing. Additional file 2:
**Input form of the VariantDB galaxy integration.** Supported VCF sources are GATK Unified Genotyper, GATK Haplotype Caller, GATK MuTect and Sam Tools VarScan. Private VariantDB servers can be added in the tool configuration. Additional file 3:
**Alternative output format of VariantDB.** In this format, all selected annotations are presented on a single line per variant. Annotations are grouped if they represent multiple entries for the same variant (for example, alternative transcripts, multiple entries in ClinVar).

## Abbreviations

GATK: Genome Analysis Toolkit
IGV: Integrative Genomics Viewer
MAF: minor allele frequency
NGS: next generation sequencing
SNP: single-nucleotide polymorphism
WES: whole exome sequencing
