# Supplementary figures and images for: VariantDB: a flexible annotation and filtering portal for next generation sequencing data
Source: Genome Med. 2014 Oct 2;6(10):74. doi: 10.1186/s13073-014-0074-6 (PMC4210545; doi:10.1186/s13073-014-0074-6)

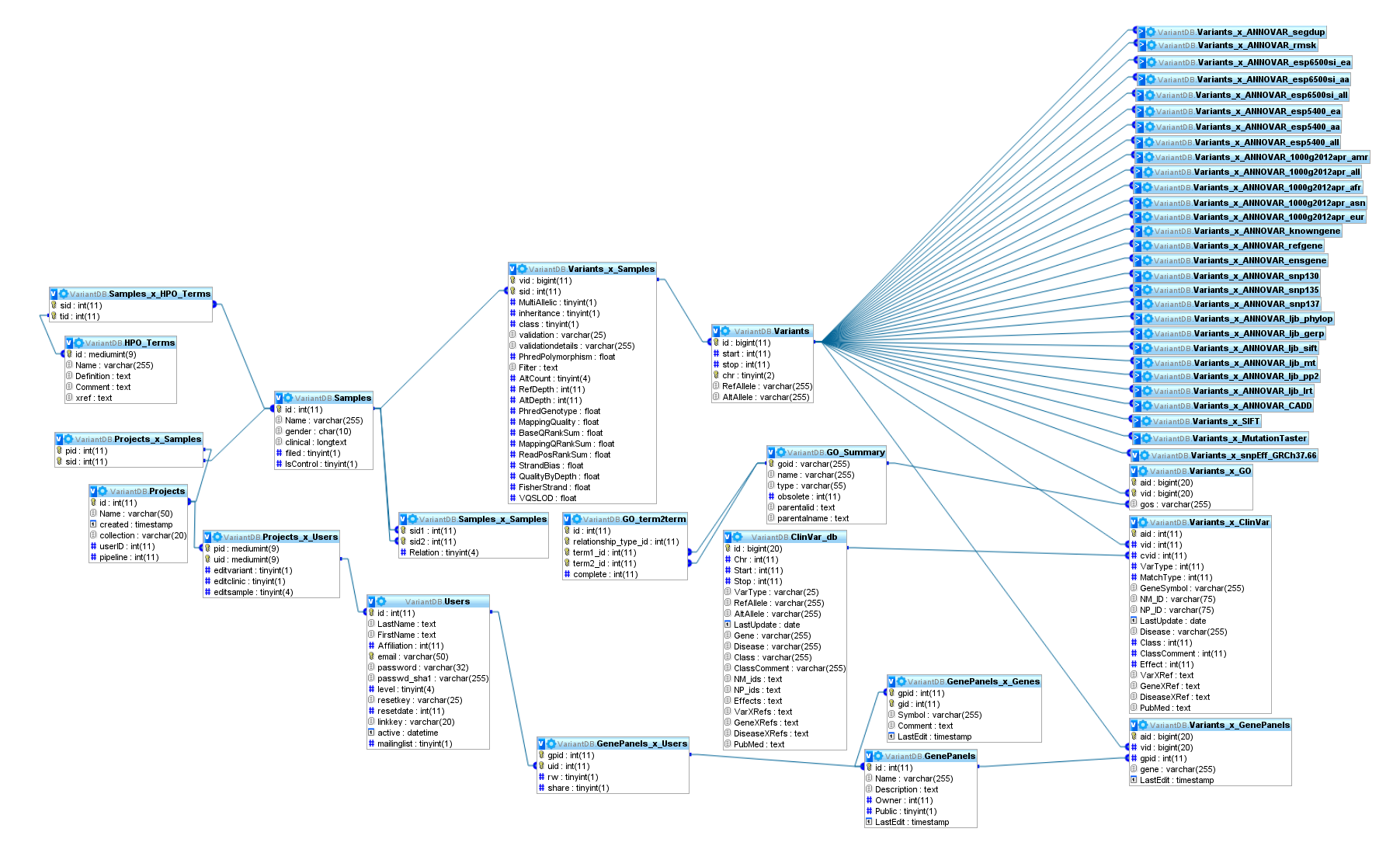

Supplement: Additional file 1: — Database layout of VariantDB. The variant table holds unique IDs and information on genomic position and allelic composition of each observed variant. Annotations are stored in separate tables linked on the variant_id. To increase performance, ClinVar and Gene Ontology are stored as summary representations of the full releases. User authentication is based on email and password combinations. Data access is controlled on the project level, with access definitions for variant-level, sample-level and phenotype-level editing. [file 13073_2014_74_MOESM1_ESM.tiff]

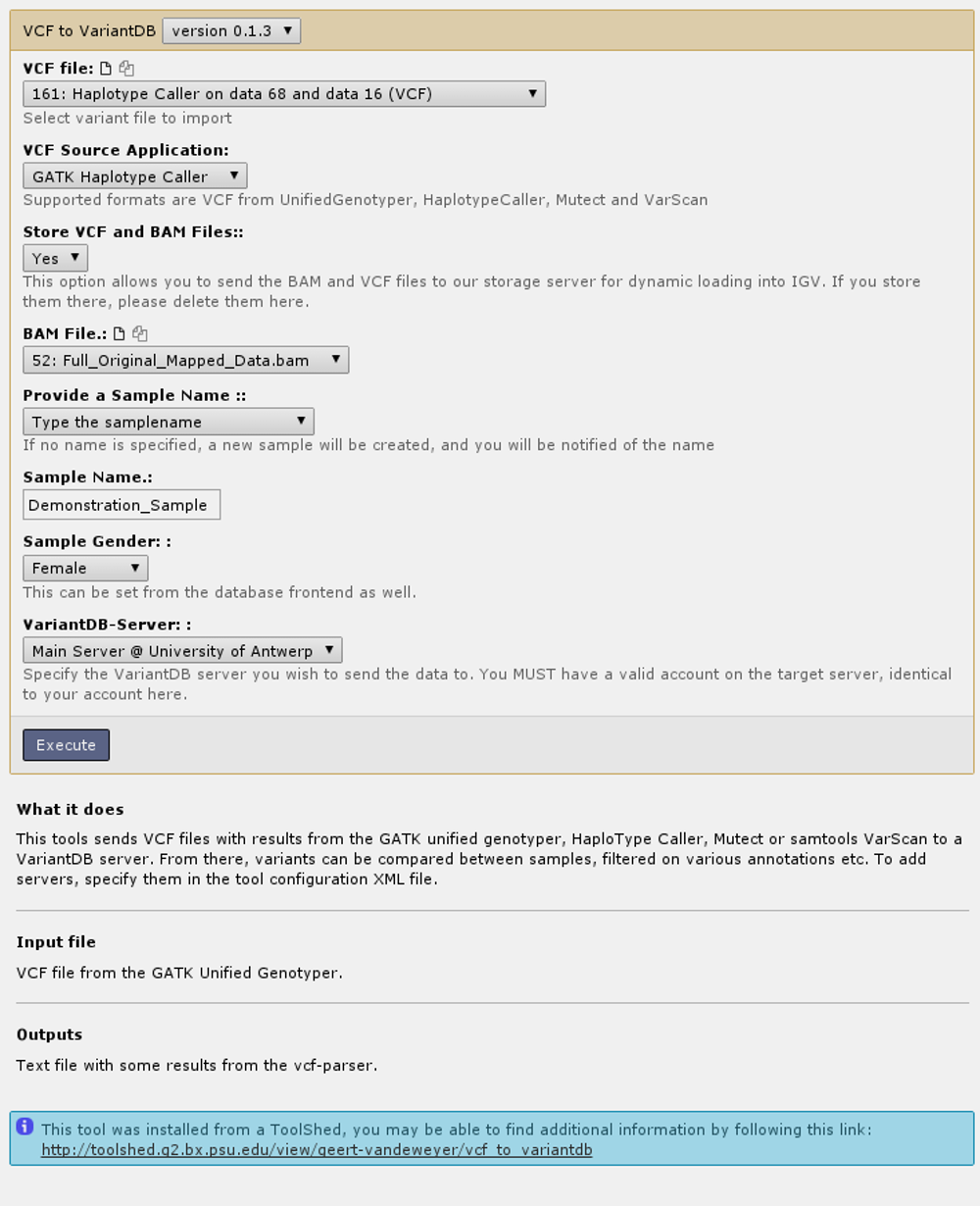

Supplement: Additional file 2: — Input form of the VariantDB galaxy integration. Supported VCF sources are GATK Unified Genotyper, GATK Haplotype Caller, GATK MuTect and Sam Tools VarScan. Private VariantDB servers can be added in the tool configuration. [file 13073_2014_74_MOESM2_ESM.tiff]

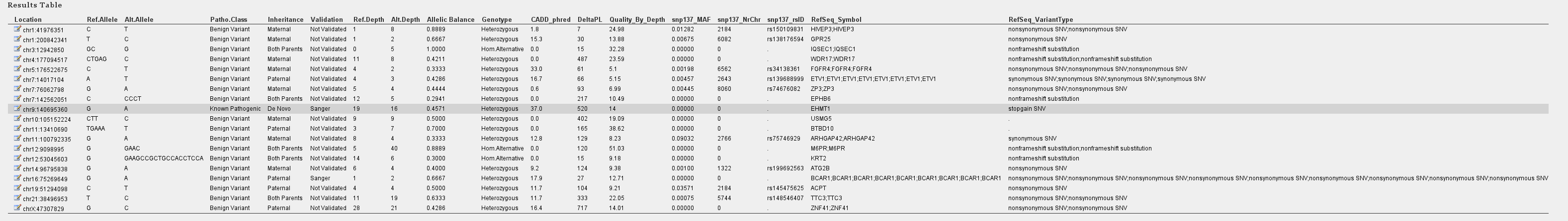

Supplement: Additional file 3: — Alternative output format of VariantDB. In this format, all selected annotations are presented on a single line per variant. Annotations are grouped if they represent multiple entries for the same variant (for example, alternative transcripts, multiple entries in ClinVar). [file 13073_2014_74_MOESM3_ESM.tiff]
